# Association Between Exposure to Age Discrimination and Nutritional Risk: Findings from a Nationwide Sample of Older Adults in South Korea

**DOI:** 10.3390/nu17233643

**Published:** 2025-11-21

**Authors:** Seong-Uk Baek, Jin-Ha Yoon

**Affiliations:** 1 Graduate School, Yonsei University College of Medicine, Seoul 03722, Republic of Korea; 2 Department of Occupational & Environmental Medicine, Severance Hospital, Seoul 03722, Republic of Korea; 3 The Institute for Occupational Health, Yonsei University College of Medicine, Seoul 03722, Republic of Korea

**Keywords:** ageism, diet, discrimination, food, nutrition

## Abstract

Background: Ageism and age-related discrimination are growing public health concerns that can have detrimental effects on the health of older adults. However, their association with nutritional health remains unexplored. This study explored the association between age discrimination and nutritional risk among older adults in Republic of Korea. Methods: This study is a cross-sectional analysis of the 2023 National Survey of Older Korean. 9951 adults aged ≥ 65 years from the 2023 National Survey of Older Koreans. Age discrimination was assessed based on the reported experiences in six everyday settings: public transportation; commercial facilities; public institutions; healthcare facilities; workplaces; and family. Nutritional status was measured using the Nutrition Screening Initiative and categorized into low; moderate; and high nutritional risk groups. Multinomial logistic regression models were used to determine the association between age discrimination and nutritional risk and estimate odds ratios and 95% confidence intervals (CIs). Results: The mean age of participants was 74.0 years. Among the participants, 73.7% reported no age discrimination, whereas 15.4%, 6.0%, and 4.9% experienced age discrimination in one; two; and three or more settings, respectively. After adjusting for socio-demographic characteristics and health factors, compared to individuals who did not experience age discrimination, those who experienced discrimination in one, two, or three or more dimensions had 1.40-fold (95% CI: 1.17–1.69), 1.46-fold (95% CI: 1.13–1.89), and 1.89-fold (95% CI: 1.44–2.48) higher odds of being at high nutritional risk, respectively. Conclusion: Age discrimination exposure may be a risk factor for poor nutritional health in older adults. Policy efforts are needed to reduce age-related discrimination and stigmatization and promote equitable conditions for healthy aging.

## 1. Introduction

Ageism refers to stigmatization and discrimination based on age [1]. This often involves the stereotyping of older adults as physically frail, mentally diminished, and less active, leading to their exclusion and unequal treatment. Given the rapid aging of the global population, age discrimination has emerged as a public health concern [2]. In recent years, age discrimination has been recognized as an important social determinant of health [3]. There is an urgent need to address prejudice, stereotypes, and discrimination against older adults.

The World Health Organization (WHO) has reported that ageism imposes a significant health burden and is associated with mental health problems, cognitive decline, and premature mortality [4]. From the perspective of the social determinants of health, preventing discrimination can contribute to improvements in both physical and mental health as well as healthcare access among older adults [2,3]. According to the healthy aging model proposed by the WHO, healthy aging is not merely about extending life expectancy but is achievable through the maintenance of physical, psychological, and social well-being [5]. Therefore, mitigating age discrimination is crucial for fostering equitable conditions that support healthy aging [6].

Age discrimination has been associated with various adverse health outcomes. For instance, exposure to age discrimination is associated with higher risks of poor subjective health, cardiovascular diseases, and depressive symptoms among older adults in the United Kingdom [7]. A prospective cohort study revealed that age discrimination was associated with an increased risk of frailty progression and development in older adults [8]. Other studies on older adults in Republic of Korea have revealed that age discrimination was linked to depressive symptoms and suicidal ideation and attempts [9,10]. Furthermore, age discrimination can compromise the quality of healthcare that older adults receive by limiting access and hindering effective care management [11].

In recent years, there has been increasing academic interest to elucidate the social determinants of nutritional status [12,13]. Various forms of discrimination, including those based on gender, sexual orientation, and race, can adversely affect food security and nutritional status [14,15,16]. Various mechanisms may underlie the relationship between discrimination exposure and nutritional risks. For instance, exposure to discrimination can induce social isolation and financial instability [17,18], which in turn may act as a barrier to accessing adequate food and establishing healthy dietary habits. Additionally, individuals who experience discrimination may engage in unhealthy eating habits as a coping mechanism for psychological stress [19].

Older adults are a vulnerable population with a high prevalence of poor nutritional status [20]. While various factors, such as financial poverty, social isolation, and poor mental health, are established risk factors for nutritional risk among older adults [20], the association between age discrimination and nutritional risk among older individuals remains poorly understood. To our best knowledge, no prior studies have analyzed the link between age discrimination and nutrition risk using a validated screening tool in a nationwide sample. Investigating this association could provide novel insights into the pathways through which age discrimination contributes to adverse health outcomes. Moreover, it can provide information to guide the implementation of policies aimed at enhancing the nutritional well-being of older adults. Therefore, this study aimed to examine the relationship between age discrimination and nutritional risk among older individuals in Republic of Korea.

## 2. Materials and Methods

### 2.1. Study Sample

This study is a cross-sectional, secondary data analysis using the 2023 National Survey of Older Koreans (NSOK) dataset. The NSOK is a nationwide survey that included older adults aged ≥ 65 years that is conducted in 2023. The target population is adult aged ≥ 65 years living in Republic of Korea in 2023. The 2023 NSOK was conducted by the Korea Institute for Health and Social Affairs (KIHASA). Trained interviewers administered the surveys via the household-visiting method from September to November 2023. The NSOK employed a multistage systematic sampling method to recruit a nationally representative sample of approximately 10,000 older adults living in Republic of Korea [18]. The 2023 NSOK was approved by the Institutional Review Board of the KIHASA (No. 2023-078). The protocol of study was approved by the Institutional Review Board of the Yonsei University Health System (No. 4-2025-0337). Written informed consent was obtained from all participants. This study followed the STROBE guideline (Supplementary Details).

**Figure 1:**
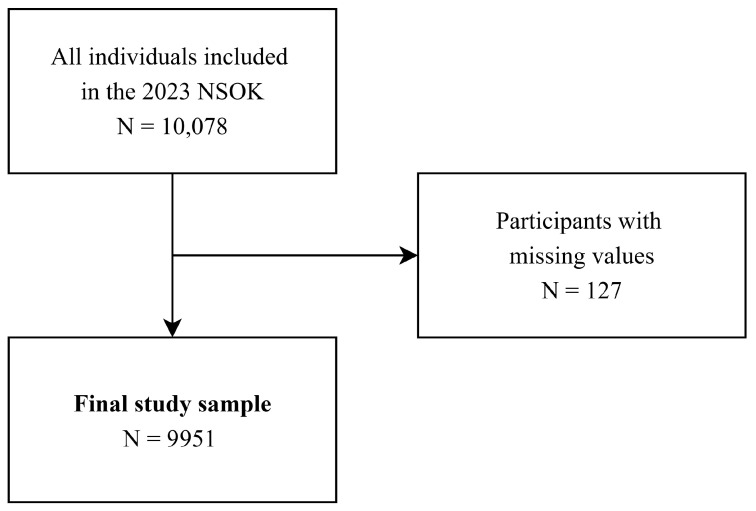
Flowchart of the study sample selection.

A flowchart of the study sample selection procedure is shown in Figure 1. Initially, 10,078 older adults were included in the 2023 NSOK. Among them, 127 (1.3%) with missing data were excluded. As only 1.3% of the participants had missing values, the listwise deletion was conducted to obtain the final sample. Consequently, the final sample comprised 9951 individuals. Raw data are available at https://mdis.kostat.go.kr (accessed on 22 July 2025).

### 2.2. Age Discrimination

Exposure to age discrimination was assessed across six daily-life dimensions using the following question: “In the past year, have you ever felt ignored or discriminated against because of your age in the following situations?” The six domains included “(a) public transportation, (b) commercial facilities (e.g., restaurants, cafés, supermarkets, department stores), (c) public institutions (e.g., community centers, district offices), (d) healthcare facilities (e.g., hospitals, clinics), (e) workplace, and (f) family.” For each dimension, participants indicated their experience of discrimination as “not applicable/no” or “yes.” Respondents who answered “yes” to a given domain were considered exposed to age discrimination in that context. The Cronbach’s alpha was 0.64 in the sample. Next, the number of age discrimination dimensions, ranging from 0 to 6, was calculated. Individuals were categorized into those who experienced no discrimination in any domain and those who experienced discrimination in one, two, or three or more domains to assess the dose–response association across multiple domains of nutritional risk. Because the prevalence of participants who experienced discrimination in four or more domains was low (<2%), they were combined into the “three or more domains” category.

### 2.3. Nutritional Risk

Nutritional risk was assessed using the Nutrition Screening Initiative (NSI) questionnaire, a validated tool for evaluating nutritional health status among older adults in Republic of Korea [21,22]. The NSI comprises 10 items pertaining to the indicators of poor nutritional health, each of which is answered in a dichotomous (yes/no) format. A predetermined weight ranging from 1 to 4 was assigned to each affirmative response [21]. These weighted responses were summed to create a composite NSI score with a possible range of 0 to 21. Based on the composite NSI score, nutritional status was categorized as follows: 0–2, low nutritional risk; 3–5, moderate nutritional risk; and 6–21, high nutritional risk” [21]. The 10-item NSI checklist has been validated in the older adults in Republic of Korea [22,23].

### 2.4. Covariates

The selection of covariates was based on the previous literature on the association between exposure to discriminations and health outcomes [14,15,16,19,24]. Sex (male or female) and age (continuous, in years) were included as covariates. Educational attainment was categorized as no education, elementary school, middle school, high school, and college education or higher. Income level was classified into quintiles (lowest, low, medium, high, and highest) based on the total household income. Employment status was categorized as either employed or unemployed. Functional limitations were defined as “yes” or “no” based on the Activities of Daily Living and Instrumental Activities of Daily Living assessments. Depressive symptoms were defined as “yes” for Short Geriatric Depression Scale scores ≥ 6 and “no” otherwise. Cognitive performance was assessed using the Korean version of the Mini-Mental State Examination, 2nd edition. Body mass index (BMI) (kg/m^2^) was calculated from the measured weight and height. The inclusion of covariates was determined a priori based on the theoretical relationship between the exposure, outcome, and potential confounders.

### 2.5. Statistical Analyses

The distribution of characteristics among the samples was examined according to the age discrimination categories. For the main analyses, multinomial logistic regression models were used to examine the association between age discrimination and nutritional risk categories, with “low nutritional risk” as the reference category. Both unadjusted and fully adjusted models were sequentially fitted, and the associations were presented as odds ratios (ORs) with 95% confidence intervals (CIs). Subsequently, the independent associations between each of the six age discrimination domains and nutritional risk were investigated. For sensitivity analysis, linear regression models were fitted using the continuous NSI score as the dependent variable. All analyses were conducted using the R software (version 4.5.0). The R package “VGAM” and its function “vglm” were used to perform multinomial logistic regression analyses. To ensure the sample accurately reflects the target population, survey weights were calculated by region, sex, and age group. Survey weights applied to all regression models to enhance the generalizability of the findings. For all regression models, the absence of multi-collinearity was confirmed based on the variance inflation factor less than 5. For model diagnostics, Akaike Information Criterion values were reported for each regression model.

## 3. Results

The characteristics of the study participants are shown in Table 1. Among the participants, 73.7% reported no age discrimination, whereas 15.4%, 6.0%, and 4.9% experienced age discrimination in one, two, and three or more dimensions, respectively. The proportion of individuals with high nutritional risk was 11.1% among those who did not experience age discrimination and 14.9%, 16.6%, and 21.3% among those who experienced age discrimination in one, two, and three or more dimensions, respectively.

Table 2 and Table S1 show the association between age discrimination and nutritional risk in the study sample using multinomial logistic regression models. Compared with older adults who reported no age discrimination, those experiencing discrimination in one dimension had higher odds of moderate (OR = 1.17, 95% CI: 1.04–1.32) and high nutritional risk (OR = 1.40, 95% CI: 1.17–1.69). Similarly, discrimination in two dimensions was associated with increased odds of moderate (OR = 1.20, 95% CI: 1.00–1.43) and high nutritional risk (OR = 1.46, 95% CI: 1.13–1.89). The strongest associations were observed among those experiencing discrimination in three or more dimensions, with ORs of 1.53 (95% CI: 1.25–1.88) for moderate nutritional risk and 1.89 (95% CI: 1.44–2.48) for high nutritional risk.

**Figure 2:**
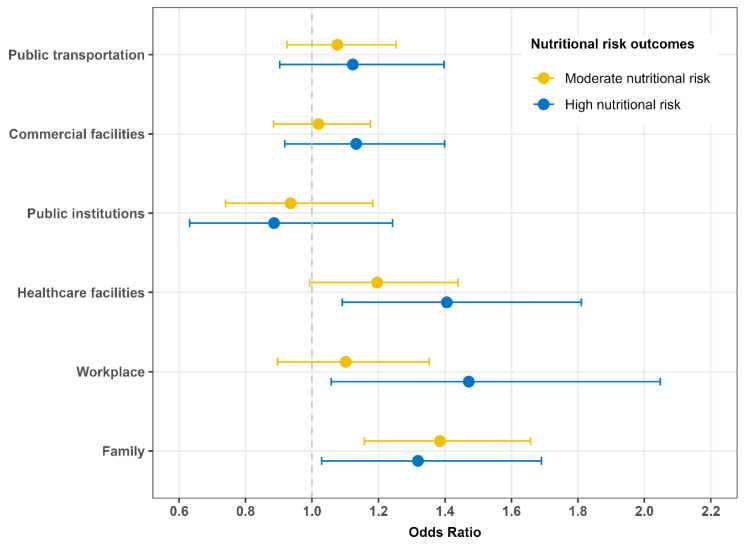
Independent associations between dimensions of age discrimination and nutritional risk in the multinomial logistic regression model (reference outcome: low nutritional risk). All models were adjusted for sex, age, educational level, income level, employment status, functional limitations, depressive symptoms, cognitive performance, and body mass index.

The independent associations between each age-discrimination dimension and nutritional risk are shown in Figure 2 and Table S2. Exposure to age discrimination in healthcare facilities, workplace, and family was associated with 1.41-fold (95% CI: 1.09–1.81), 1.47-fold (95% CI: 1.06–2.05), and 1.32-fold (95% CI: 1.03–1.69) higher odds of high nutritional risk, respectively.

Sensitivity analysis (Table S3) showed that, compared with those not experiencing age discrimination, individuals exposed to discrimination in one, two, and three or more dimensions had 0.14-point (95% CI: 0.04–0.25), 0.35-point (95% CI: 0.19–0.50), and 0.43-point (95% CI: 0.26–0.59) higher NSI scores, respectively. The results based on the probability difference scale are presented in Table S4.

## 4. Discussion

This study examined the relationship between age discrimination exposure and nutritional risk among older adults in Republic of Korea. The findings show that compared with those not exposed to age discrimination, older adults experiencing age discrimination are more likely to have a high nutritional risk. Furthermore, the prevalence of age discrimination was notably higher than that reported in the previous study, which indicated a prevalence of approximately 3% among those aged 65 and over in the European Social Survey 2021 data [25]. Therefore, this study highlights the need to address age discrimination in older adults and promote nutritional health.

This finding suggests an urgent need for intensified efforts to reduce age discrimination in Republic of Korea. The findings of this study corroborate those of previous studies, showing that exposure to various forms of discrimination is linked to poor nutrition. For instance, perceived race discrimination can negatively impact eating habits and food security [16,26]. Discrimination and harassment against individuals who identify as sexual minorities have been linked to food insecurity and eating disorders [15,27,28]. Additionally, perceived discrimination was associated with an increased likelihood of food insecurity among migrants in Peru [29]. A study of Israelis aged ≥ 50 years found that those who age discrimination had lower vegetable consumption than those who did not, which may a potential mechanism linking discrimination to poor self-rated health and chronic conditions [30].

Complex mechanisms may underlie the association between exposure to age discrimination and high nutritional risk among older adults. First, ageism or age discrimination can exclude older adults from various social roles and activities, leaving them with fewer social interactions and fostering feelings of loneliness and social exclusion [31,32,33]. Social networks and connectedness are critical for supporting healthy nutrition; for instance, social support facilitates older adults’ selection and consumption of healthy foods and helps them secure adequate food through access or assistance from others when they are ill or financially constrained [34,35]. In contrast, individuals experiencing social isolation and loneliness are more likely to engage in harmful eating habits such as low fruit and vegetable intake, hazardous alcohol consumption, and poor dietary quality [36]. Second, biopsychosocial pathways that link exposure to discrimination with poor nutrition have been identified, where discrimination is a significant psychosocial stressor that can adversely influence eating behaviors. Chronic stress induced by discrimination can influence neural activity in the prefrontal regions involved in impulse regulation and limbic regions associated with reward processing, thereby modulating appetitive responses [19,37,38]. Additionally, exposure to discrimination has been liked to alterations in the hypothalamic–pituitary–adrenal axis, influencing appetite and nutritional behaviors [39]. From a geroscience perspective, chronic stress has been reported to adversely affect healthy eating choices, thereby undermining metabolic and nutritional resilience. [40,41]. Third, age discrimination can lead to loss of autonomy, resulting in diminished control over food choices and consumption. This study specifically found that discrimination experienced across key settings, including healthcare facilities, workplace, and family, was significantly associated with high nutritional risk. For instance, the specific nutritional needs of older adults may receive insufficient attention from healthcare providers, particularly in settings where age discrimination is prevalent [30,42]. Furthermore, this loss of autonomy can extend to professional or domestic environments, where they may experience diminished decision-making power regarding food selection and meal consumption, which can contribute to poor nutritional status. Fourth, the findings of this study should be interpreted in light of the cultural context of Republic of Korea, which is deeply influenced by Confucianism. Confucianism, prevalent throughout East Asia, emphasizes respect for older adults [43]. However, in recent years, societal changes such as modernization, rapid technological advancement, and globalization have contributed to the marginalization of older adults and age discrimination. Such marginalization may further intensify the psychological stress associated with age discrimination among older adults and affect their coping responses and nutritional behaviors.

This study underscores the need for active policy efforts aimed at mitigating ageism and age discrimination toward older adults. For effective intervention, multi-dimensional policy efforts are required to reduce age discrimination against older adults, which should comprehensively encompass various settings, including household, workplace, and healthcare services [44]. Furthermore, the implementation of policies designed to enhance food security and promote healthy eating practices is needed to improve overall nutritional status of the older adults.

This study had some limitations. First, the causal impact of age discrimination on nutritional risk among older adults could not be established, given the cross-sectional nature of this study. A potential reverse causality must also be considered; individuals with a worse nutritional status may experience increased frailty, which in turn could increase their susceptibility to age discrimination [45]. Therefore, the findings of this study should be interpreted as indicating an association between age discrimination and nutritional status, rather than a causal relationship. Future studies should clarify the temporal and causal relationships between age discrimination and nutritional risk within a longitudinal framework. Second, the variables were susceptible to measurement errors as they were self-reported. For instance, recall bias or individuals’ reluctance to disclose discrimination experiences may result in inaccurate measurements. In addition, it should be acknowledged that perceptions of discrimination sensitivity may vary across individuals. Third, several important factors, such as information on individuals’ eating disorders, social networks and support, distance to facilities such as grocery stores or restaurants, and the dietary habits of cohabiting family members, were not collected, although they may have influenced the association between the two variables. Fourth, given that cultural background can substantially influence both societal ageism and dietary habits, our findings may not necessarily be generalizable to older adults in other cultural or regional contexts. For example, society of the Republic of Korea is deeply rooted in Confucian culture, which emphasizes a hierarchy based on age [43]. The manifestations of age discrimination in East Asia may differ significantly from those found in Western cultures. Fifth, the potential residual confounding should be considered. For instance, this study could not encompass factors such as socioeconomic status, social support, household composition, accessibility to foods, and comorbidities, were not addressed due to the lack of information. Sixth, our study lacked detailed information on the actual intake of specific macro- and micronutrients. In addition, although age discrimination was assessed across multiple domains, the specific forms or manifestations of such discrimination could not be identified. Therefore, future studies should employ more in-depth assessments to capture the nuanced aspects of both nutrient intake and discrimination experiences.

However, this study had several strengths. First, to the best of our knowledge, this study is one of the few to demonstrate an association between age discrimination exposure and nutritional risk. These observations provide novel insights for policy efforts aimed at reducing ageism and age discrimination while promoting the nutritional health of older adults. Second, the analyses were conducted using a nationally representative sample of older individuals, which enhanced the generalizability of the findings.

## 5. Conclusions

This study revealed that exposure to age discrimination was associated with higher nutritional risk among older adults in Republic of Korea. Particularly, the association with nutritional risk was stronger among those exposed to multiple dimensions of age discrimination. Policymakers should consider implementing multidimensional interventions to reduce age discrimination across households, workplaces, and the social sphere. For clinicians, monitoring and supporting the nutritional status of individuals who experience age discrimination may be warranted.

## Figures and Tables

**Table 1:** Characteristics of the study participants.

	Overall	Dimensions of Age Discrimination Experienced			
		None	One	Two	Three or More
	*n* = 9951	*n* = 7333	*n* = 1537	*n* = 598	*n* = 483
Sex					
Male	3824 (38.4%)	2807 (38.3%)	583 (37.9%)	238 (39.8%)	196 (40.6%)
Female	6127 (61.6%)	4526 (61.7%)	954 (62.1%)	360 (60.2%)	287 (59.4%)
Age (years)					
Mean (SD)	74.0 (6.8)	74.2 (6.7)	73.6 (6.7)	73.7 (6.8)	73.6 (7.1)
Educational level					
No education	1435 (14.4%)	1088 (14.8%)	208 (13.5%)	76 (12.7%)	63 (13.0%)
Elementary school	2920 (29.3%)	2212 (30.2%)	412 (26.8%)	175 (29.3%)	121 (25.1%)
Middle school	2114 (21.2%)	1562 (21.3%)	332 (21.6%)	133 (22.2%)	87 (18.0%)
High school	2860 (28.7%)	1977 (27.0%)	515 (33.5%)	185 (30.9%)	183 (37.9%)
College or higher	622 (6.3%)	494 (6.7%)	70 (4.6%)	29 (4.8%)	29 (6.0%)
Income level					
Lowest	2107 (21.2%)	1699 (23.2%)	250 (16.3%)	83 (13.9%)	75 (15.5%)
Low	2167 (21.8%)	1645 (22.4%)	321 (20.9%)	116 (19.4%)	85 (17.6%)
Medium	2026 (20.4%)	1467 (20.0%)	335 (21.8%)	119 (19.9%)	105 (21.7%)
High	1910 (19.2%)	1384 (18.9%)	317 (20.6%)	121 (20.2%)	88 (18.2%)
Highest	1741 (17.5%)	1138 (15.5%)	314 (20.4%)	159 (26.6%)	130 (26.9%)
Employment status					
Employed	3942 (39.6%)	2846 (38.8%)	634 (41.2%)	257 (43.0%)	205 (42.4%)
Unemployed	6009 (60.4%)	4487 (61.2%)	903 (58.8%)	341 (57.0%)	278 (57.6%)
Functional limitation					
Yes	1648 (16.6%)	1087 (14.8%)	308 (20.0%)	125 (20.9%)	128 (26.5%)
No	8303 (83.4%)	6246 (85.2%)	1229 (80.0%)	473 (79.1%)	355 (73.5%)
Depressive symptoms					
Yes	7893 (79.3%)	6021 (82.1%)	1149 (74.8%)	422 (70.6%)	301 (62.3%)
No	2058 (20.7%)	1312 (17.9%)	388 (25.2%)	176 (29.4%)	182 (37.7%)
K-MMSE~2					
Mean (SD)	24.5 (4.8)	24.8 (4.4)	23.6 (5.8)	23.7 (5.2)	23.6 (5.0)
Body mass index					
Mean (SD)	23.6 (2.6)	23.7 (2.7)	23.6 (2.5)	23.4 (2.5)	23.7 (2.5)
Nutritional risk					
Low risk	4243 (42.6%)	3266 (44.5%)	602 (39.2%)	220 (36.8%)	155 (32.1%)
Moderate risk	4463 (44.8%)	3253 (44.4%)	706 (45.9%)	279 (46.7%)	225 (46.6%)
High risk	1245 (12.5%)	814 (11.1%)	229 (14.9%)	99 (16.6%)	103 (21.3%)

K-MMSE~2, Korean version of the Mini-Mental Status Examination, 2nd version; SD, standard deviation.

**Table 2:** Association between exposure to age discrimination and nutritional risk among older adults (reference outcome: low nutritional risk).

	Unadjusted Model		Fully Adjusted Model	
	Nutritional Risk Outcomes		Nutritional Risk Outcomes	
	Moderate Risk	High Risk	Moderate Risk	High Risk
	OR (95% CI)	OR (95% CI)	OR (95% CI)	OR (95% CI)
**Age discrimination**				
None	Reference	Reference	Reference	Reference
One dimension	1.12 (1.00–1.26)	1.50 (1.28–1.77)	1.17 (1.04–1.32)	1.40 (1.17–1.69)
Two dimensions	1.16 (0.98–1.38)	1.79 (1.43–2.25)	1.20 (1.00–1.43)	1.46 (1.13–1.89)
Three or more dimensions	1.51 (1.25–1.82)	2.67 (2.11–3.39)	1.53 (1.25–1.88)	1.89 (1.44–2.48)

OR, odds ratio; CI, confidence interval. The adjusted models controlled for sex, age, education level, income level, employment status, functional limitations, depressive symptoms, cognitive performance, and body mass index.

## Data Availability

The data are accessible at https://mdis.kostat.go.kr (accessed on 22 July 2025).

## Supplementary Materials

The following supporting information can be downloaded at: https://www.mdpi.com/article/10.3390/nu17233643/s1, Table S1: Association between exposure to age discrimination and nutritional risk among older adults (reference outcome: low nutritional risk); Table S2: Association between exposure to age discrimination and nutritional risk among older adults (reference outcome: low nutritional risk); Table S3: Association between exposure to age discrimination and NSI scores on linear regression models; Table S4: Age discrimination and nutritional risk based on the probability difference scale.
